# The Treatment of Burns

**Published:** 1907-09

**Authors:** Frank K. Boland

**Affiliations:** Atlanta, Ga.


### THE TREATMENT OF BURNS.

By Frank K. Boland, M. D.

---

The primary treatment of burns is planned to combat pain, shock and infection. A proper surgical dressing does much to relieve the pain, though in severe cases hypodermic medication is indicated. Shock is treated as in other conditions, while the prevention of infection depends upon the cleanliness of the first dressing.

The best remedy for shock has been shown by Crile and others to be adrenalin chloride, given hypodermically (gttx of 1:1000 solution), or better in normal salt solution (ʒ 1 of 1:1000 sol. to 1 quart), subcutaneously, or per rectum, or in desperate cases half the quantity intravenously.

It is well to remember that a burnt area is apt to be sterile on account of the great heat which has produced it, and if infection be not added to the wound by unclean handling, much will have been done toward hastening our patient's recovery and lessening the amount of cicatrix which may result. To this end, when possible, instruct those who first see the patient to cover the wound with only the cleanest cloths. More than a local infection may be absorbed through a burn. Cases of tetanus have been reported from covering burnt areas with horse blankets. Of course the patient must be kept warm to avoid shock: and protecting the wound from the air undoubtedly dimin- minishes the pain.

If necessary to remove the clothing it is best to cut it and remove carefully. If the clothing is adherent to the skin, soak thoroughly with soda bicarb. solution or saline. In extensive burns one of the best things to be done with the sufferer is to put him in a bath-tub of warm water, which is frequently changed and to which soda bicarb. is added, and there soak off his clothing and keep him in it for twenty-four hours or longer.

The skin adjacent to a burn should be made clean by the usual methods, care being taken that no strong chemical solutions come in contact with the burnt area, on account of the liability to absorption. The injured part should be cleansed gently with boric acid or saline solution, and bits of necrotic tissue removed. It is not best to open blebs at the first dressing, since it only opens another avenue for infection. The second dressing is time enough for this. Only an opening should be made large enough to empty the serum. The collapsed bleb falls down as a skin graft. Anæsthesia is often necessary for a thorough cleansing.

A one per cent aqueous solution of picric acid, applied on sterile lint or gauze, is the best first dressing for burns. It alleviates the pain and hastens healing as no other remedy does. A great advantage over carron oil is in its cleanliness. The yellow stain may be removed by ammonia, alcohol or oxalic acid. After the dressing is applied do not let it get dry, but keep moist with the same solution, one-half strength. In extensive burns of the extremities the use of splints makes the patient more comfortable and reduces the amount of flexure which may occur.

The dressing should not be changed oftener than everv 48 hours unless infection has taken place. In this case the treatment must follow lines for infection under other conditions, for which moist antiseptic dressings are usually best. It must be borne in mind that a burnt surface absorbs readily and strong chemical solutions are dangerous. A few cases of picric acid poisoning have been reported, but such an accident is scarcely to be feared. The wound is kept clean with mild antiseptic solutions, and picric acid reapplied until healing takes place, or granulation commences, when usually some ointment is indicated. Oxide of zinc is simple, and as effective as any.

If sranulaaire is slow or exuberant, nitrate of silver is indicated, and if this does not heal promptly, no time should be lost in resorting to skin grafting. On flexor surface, especially near joints, when there is a tendency to scar formation and contracture, skin-grafting should be done early.

resorting to skin grafting. On flexor surfaces, especially near

